# Induction of Apoptosis in MDA-MB-231 Cells Treated with the Methanol Extract of Lichen *Physconia hokkaidensis*

**DOI:** 10.3390/jof7030188

**Published:** 2021-03-05

**Authors:** Ji-In Noh, Seul-Ki Mun, Eui Hyeon Lim, Hangun Kim, Dong-Jo Chang, Jae-Seoun Hur, Sung-Tae Yee

**Affiliations:** 1 Department of Pharmacy, Sunchon National University, Jungang-Ro, Suncheon 549-742, Korea; nji8009@naver.com (J.-I.N.); motomoto1210@naver.com (S.-K.M.); sksms147zld@naver.com (E.H.L.); hangunkim@sunchon.ac.kr (H.K.); djchang@scnu.ac.kr (D.-J.C.); 2 Department of Environmental Education, Korea Lichen Research Institute, Sunchon National University, Suncheon 549-742, Korea; jshur1@sunchon.ac.kr

**Keywords:** triple-negative breast cancer (TNBC), MDA-MB-231, MCF-7, apoptosis, anticancer, lichen, *Physconia hokkaidensis*

## Abstract

*Physconia hokkaidensis* methanol extract (PHE) was studied to identify anticancer effects and reveal its mechanism of action by an analysis of cytotoxicity, cell cycles, and apoptosis biomarkers. PHE showed strong cytotoxicity in various cancer cells, including HL-60, HeLa, A549, Hep G2, AGS, MDA-MB-231, and MCF-7. Of these cell lines, the growth of MDA-MB-231 was concentration-dependently suppressed by PHE, but MCF-7 was not affected. MDA-MB-231 cells, triple-negative breast cancer (TNBC) cells, do not express estrogen receptor (ER), progesterone receptor (PR), and human epidermal growth factor receptor 2 (HER-2), whereas MCF-7 cells are ER-positive, PR-positive, and HER-2-negative breast cancer cells. The number of cells in sub-G1 phase was increased after 24 h of treatment, and annexin V/PI staining showed that the population size of apoptotic cells was increased by prolonged exposure to PHE. Moreover, PHE treatment downregulated the transcriptional levels of Bcl-2, AMPK, and *p*-Akt, whereas it significantly upregulated the levels of cleaved caspase-3, cleaved caspase-9, and cleaved-PARP. In conclusion, it was confirmed that the PHE exhibited selective cytotoxicity toward MDA-MB-231, not toward MCF-7, and its cytotoxic activity is based on induction of apoptosis.

## 1. Introduction

Breast cancer is the most frequent cause of cancer deaths in women around the world [1]. Breast cancer remains an unsolved problem in women’s health, with 2.1 million patients and more than 600,000 deaths worldwide in 2018 [2]. Breast cancer is the most common cancer among women in China, with an incidence of 17.07% and 278,800 new cases, ranking fifth in the causes of tumor death after lung, gastric, liver, and colorectum cancers [3]. Treatment options for breast cancer include surgery, radiation therapy, chemotherapy, and targeted therapies in clinical treatments [4]. However, cancer metastasizes or relapses due to drug resistance and toxicity [5]. Therefore, exploring novel therapeutics against breast cancer would benefit public health. It is common for breast cancer patients to highly express female hormone receptors, including estrogen receptor (ER) and progesterone receptor (PR), or/and human epidermal growth factor receptor 2 (HER-2), which are biomarkers for the treatment of breast cancer in targeted therapy [6].

Triple-negative breast cancer (TNBC) is characterized by a relatively high rate of early invasion, metastasis, and mortality among all breast cancers, accounting for 15–20% of all breast cancer patients [7]. TNBC is a subtype of breast cancer that is deficient in estrogen receptors and progesterone receptors and has low human epidermal growth factor receptor 2 (HER-2) expression, which leads to poor prognosis due to the difficulty of medication in targeted therapy [8,9,10]. Of patients with the TNBC subtype, 10–20% tend to have shorter survival times owing to high malignancy, recurrence rate, and transferability [11]. One to three years after TNBC is diagnosed, tumors can easily metastasize to internal organs, and 40% of the metastasis occurs in the lungs [12]. Therefore, the prognosis is worse than that of the other subtypes of breast cancer, and there is an urgent need to find new biomarkers and develop effective therapeutic strategies against TNBC. 

Lichens are classified as symbiotic organisms composed of a fungal partner and a photosynthetic organism such as an alga or cyanobacterium. In the symbiotic relationship, lichens often stimulate fungi to produce secondary metabolites that have important biological roles, such as self-defense against microbial infection [13,14]. Although lichens and their secondary metabolites with promising anticancer activities have been identified, their beneficial effect as an anticancer agent is not conclusive, due to a lack of evidence [14,15,16]. *Physconia* is a genus of lichenized fungi in the family Physciaceae. It grows mainly in temperate or boreal regions in North America, Europe, Asia, Africa, South America, and Australia and is found in bark, wood, rock, and soil [17].

In our preliminary study that screened four different species of lichens, assessing their effects on various cell lines, the methanol extract of *Physconia hokkaidensis* (PHE) showed the most selective cytotoxicity toward TNBC MDA-MB-231 cells compared to MCF-7 breast cancer cells, although it was not the most cytotoxic toward MDA-MB-231 cells (data not shown). Moreover, just a few studies on the biological activity of *Physconia* have been reported [18]. Hence, this study aimed to disclose the molecular mechanism of the anticancer activity of *Physconia hokkaidensis* methanol extract on MDA-MB-231 cells, the human TNBC adenocarcinoma cell line, which can provide the evidence needed to begin the successful development of a novel anticancer agent from lichen metabolites.

## 2. Materials and Methods

### 2.1. Chemicals and Reagents

Roswell Park Memorial Institute 1640 medium (RPMI-1640), fetal bovine serum (FBS), and penicillin/streptomycin solution were purchased from Hyclone Laboratories (CA, USA). Cell counting kit-8 (CCK-8) and dimethyl sulfoxide (DMSO) were obtained from Dojindo Laboratories (Kumamoto, Japan) and Sigma-Aldrich (St. Louis, MO, USA), respectively. FITC annexin V Apoptosis Detection Kit and propidium iodide (PI) were purchased from BD Biosciences (San Jose, CA, USA). Radioimmunoprecipitation assay buffer, protease and phosphatase inhibitor cocktail, BCA protein assay kit, 4–12% bis-tris plus gels, nitrocellulose membranes, TBS Tween 20 Buffer, Starting Block T20 Blocking Buffer, enhanced chemiluminescence kit, and the following antibodies: caspase-9, cleaved caspase-9, PARP, cleaved PARP, Bcl-2, β-actin, and horseradish peroxidase (HRP)-conjugated secondary antibody were acquired from Thermo Fisher (Rockford, IL, USA). Antibodies against caspase-3, cleaved caspase-3, AMPKα, Akt, and phospho-Akt were purchased from Cell Signaling (Danvers, MA, USA).

### 2.2. Collection and Preparation of the Lichen

*Physconia hokkaidensis* (*Kol.170047,* PH) was provided by Allied Bioresource Center in the Korean Lichen Research Institute, Sunchon National University, Korea. PH was collected from the bark of Gwaneumsa Temple in Jeju Island, Korea. The dried lichen thalli (60 g) was extracted with 2 L methanol (MeOH) at room temperature for 48 h using sonication. The extract was then filtered and concentrated under vacuum at 40 °C using a rotary evaporator. The dry extract powders were stored at –20 °C until further use. Phytochemical analysis of methanol (MeOH) lichen extract was performed using an HPLC–UV (SHIMADZU, LC–20A) system [19].

### 2.3. Cell Culture

Human acute promyelocytic leukemia cells (HL-60), human lung carcinoma cells (A549), human cervical adenocarcinoma cells (HeLa), human hepatoblastoma cells (Hep G2), human gastric carcinoma cells (AGS), human breast adenocarcinoma cells (MDA-MB-231 and MCF-7), and Madin–Darby canine kidney (MDCK) cells were obtained from the Korean Cell Line Bank (Seoul, Korea). The cells were cultured in RPMI-1640 medium, or Dulbecco’s Modified Eagle Medium (DMEM) for MDCK cells, containing 10% FBS, 1% penicillin/streptomycin, and 0.1% 2-mercaptoethanol. Cultures were maintained at 37 °C and 5% CO_2_, and the media were changed every two days.

### 2.4. Cytotoxicity Assay

Cell viability was determined using the CCK-8 assay. Briefly, HL-60, HeLa, A549, Hep G2, AGS, MDA-MB-231, and MCF-7 cells were resuspended in RPMI-1640, or DMEM for MDCK cells, at 3 × 10^5^ cells/mL or 5 × 10⁴ cells/mL. The cell suspension (100 µL) was added to each well of the 96-well plate and incubated for 24 h at 5% CO_2_ and 37 °C. After the incubation, 100 µL of the medium supplemented with 1, 3, 10, and 30 µg/mL of PHE was added to each well and incubated at 5% CO_2_ and 37 °C. After 24 h, 100 µL of solution was removed from each well and CCK-8 (10 µL/well) was dispensed. After 2 h, the absorbance was detected at 450 nm with a microplate reader (Versa max, Molecular Devices, CA, USA). IC_50_ values were calculated from the cell viability values at each concentration.

### 2.5. Cell Cycle Assay

MDA-MB-231 (1 × 10^5^ cells/well) and MCF-7 (5 × 10^4^ cells/well) cells were grown in 24-well plates and incubated for 24 h at 5% CO_2_ and 37 °C. After the incubation, a medium containing 1, 3, 10, and 30 µg/mL PHE was added to each well and incubated at 5% CO_2_ and 37 °C. After 24 h, the treatment medium and the attached cells were collected after centrifugation at 1200 rpm. The cells were fixed with 70% ethanol at 4 °C overnight. The cells were incubated with ribonuclease A (100 µg/mL) for 20 min at room temperature in the dark and stained with propidium iodide (50 µg/mL) for 30 min at room temperature in the dark. The DNA content of the stained cells was analyzed by flow cytometry (FACS Canto 2, BD Biosciences).

### 2.6. Annexin V/PI Staining

MDA-MB-231 (1 × 10^5^ cells/well) and MCF-7 (5 × 10^4^ cells/well) cells were grown in a 24-well plate and incubated for 24 h at 5% CO_2_ and 37 °C. After the incubation, a medium containing 1, 3, 10, and 30 µg/mL PHE was added to each well and incubated at 5% CO_2_ and 37 °C. After 48 h, the cells were harvested, washed with PBS, and stained with 5 µL of annexin V-FITC/PI (1 mg/mL). The stained cells were analyzed by flow cytometry.

### 2.7. Western Blot Assay

MDA-MB-231 cells were incubated in a six-well plate at a density of 5 × 10^5^ cells/well for 24 h. Then, 30 µg/mL of PHE was added to the cells, which were then incubated for 24 h. The cells were washed twice with cold PBS on ice and lysed in RIPA buffer containing phosphatase and protease inhibitors cocktail. The cells were centrifuged at 14,000× *g* and 4 °C for 20 min to obtain soluble proteins. The concentration of protein samples was determined using the BCA protein assay kit. First, 40 µg of protein was mixed with SDS loading buffer (×5) and then boiled for 5 min. Then, the protein samples were separated using 4–12% bis-tris plus gels and transferred to nitrocellulose membranes, which were incubated with starting Block T20 Blocking Buffer for 3 h at room temperature. The primary antibodies against caspase-9, cleaved caspase-9, PARP, cleaved PARP, Bcl-2, caspase-3, cleaved caspase-3, AMPKα, and Akt were then treated overnight at 4 °C. After washing with TBST buffer three times for 15 min, the membranes were incubated with HRP-conjugated secondary antibodies (1:5000) for 1 h at room temperature. Then, membranes were washed with TBST buffer three times for 15 min and developed with the enhanced chemiluminescence kit. The protein bands were captured and measured using a bio-imaging system (MicroChemi 4.2 Chemilumineszenz-System, Neve Yamin, Israel).

### 2.8. Statistical Analysis

Data are presented as mean ± SD of three independent experiments. Statistical differences between groups were compared using the Student’s t-test. Probability values less than 0.05 were considered significant (*p* values * *p* <0.05, ** *p* <0.01, *** *p* <0.001).

## 3. Results

### 3.1. PHE Decreases Viability of Cancer Cell Lines

**Figure 1:**
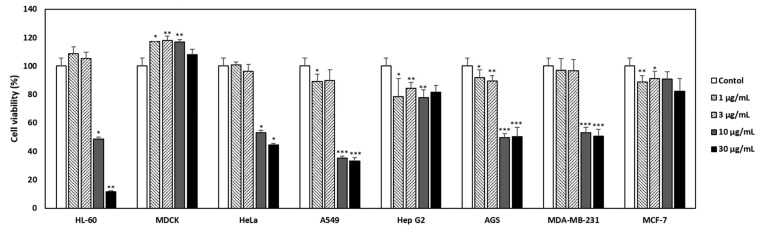
Effect of *Physconia hokkaidensis* methanol extract (PHE) on the viability of various cell lines. Seven cancer cell lines and a normal cell line were treated with the PHE (1, 3, 10, and 30 µg/mL) for 24 h. The culture supernatant was removed and cell counting kit-8 was added. Viability was quantified using a microplate reader. All data are expressed as mean ± SD of three independent experiments. * *p* < 0.05, ** *p* < 0.01, *** *p* < 0.001 compared to the control of each cell line.

The cytotoxic effects of PHE on HL-60 (a human leukemia cell), MDCK (a mammalian kidney cell), HeLa (a human cervical cancer cell), A549 (a non-small-cell lung cancer cell), Hep G2 (a human liver cancer cell), AGS (a human gastric adenocarcinoma cell), MDA-MB-231 (a TNBC breast cancer cell), and MCF-7 (a human breast cancer cells) were analyzed using CCK-8 analysis (Figure 1). Dose-dependent treatment of PME did not affect the viability of MDCK, Hep G2, and MCF-7, whereas it did demonstrate cytotoxicities on HL60, Hela, A549, and MDA-MB-231 cells. It was also not toxic in MDCK and MCF-7 when PHE was treated at a concentration of 100 µg/mL (Figure S1, Table 1). Interestingly, PHE was cytotoxic to MDA-MB-231 TNBC cells but not to MCF-7, which is ER-(+), PR-(+), and HER-2-(−) breast cancer cell. The IC_50_ values of PHE were >30 µg/mL in MCF-7 cells and 23.77 ± 1.2 µg/mL in MDA-MB-231 cells, as summarized in Table 1. Consequently, we paid attention to the selective cytotoxicity of PHE against DMA-MB-231 TNBC cells compared to MCF-7 breast cancer cells, due to the poor prognosis and limited treatment options of patients with TNBC cancer. Hence, we performed subsequent experiments to identify and validate the anticancer effect of PHE on TNBC breast cancer cells.

### 3.2. PHE Increased the Proportion of MDA-MB-231 Cells in the Sub-G1 Phase

**Figure 2:**
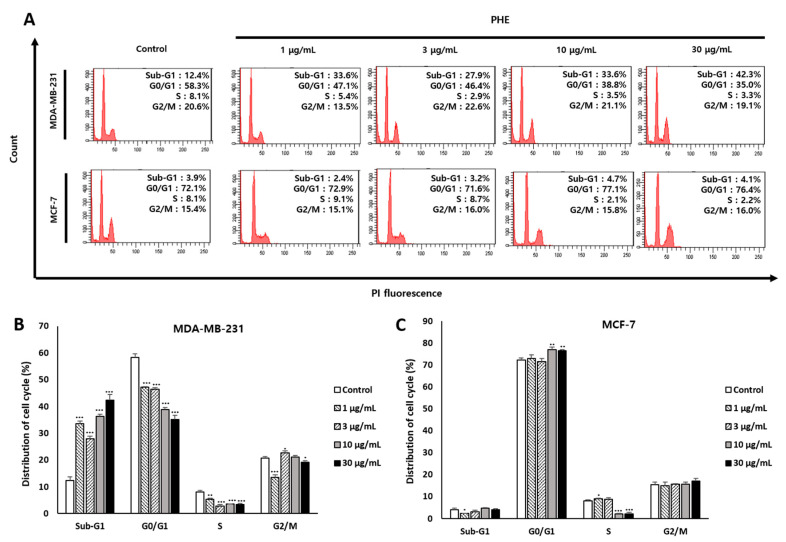
*Physconia hokkaidensis* methanol extract (PHE) induces the cell cycle arrest of MDA-MB-231 cells at the sub-G1 phase. (**A**) The cell cycle distribution of PHE-treated MDA-MB-231 and MCF-7 cells for 24 h. The cells were measured using a flow cytometer. (**B**,**C**) Statistical analysis of cell cycle distribution of PHE-treated MDA-MB-231 and MCF-7 cells. All data are expressed as mean ± SD of three independent experiments. * *p* < 0.05, ** *p* < 0.01, *** *p* < 0.001 compared to the control of each cell line.

As PHE showed noticeable cytotoxicity against MDA-MB-231 cells compared to MCF-7 cells, we first inspected the effect of PHE on cell cycle progression to understand the mode of action. To investigate whether the effect of PHE was mediated via the regulation of cell cycle progression, flow cytometric analysis was conducted on MDA-MB-231 and MCF-7 cells. PHE at 1, 3, 10, and 30 µg/mL increased the proportion of MDA-MB-231 cells in the sub-G1 cell cycle phase from 12.43 ± 1.33% observed in the control to the percentages of 33.6 ± 0.95%, 27.9 ± 1.05%, 36.33 ± 0.72%, and 42.36 ± 2.05%, respectively (Figure 2A). However, PHE treatment did not result in a significantly increased proportion of MCF-7 cells in the sub-G1 phase (Figure 2C). These data suggest that PHE leads to the reduction of viability of MDA-MB-231 by cell cycle arrest at the sub-G1 phase (Figure 2B).

### 3.3. Effects of PHE on MDA-MB-231 Cell Membrane Change

**Figure 3:**
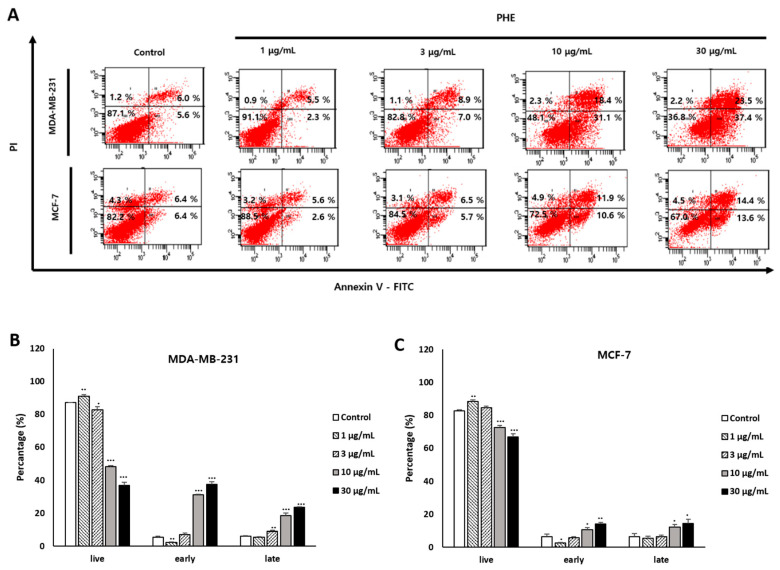
A 48-h treatment of *Physconia hokkaidensis* methanol extract (PHE) induces MDA-MB-231 cell membrane change. (**A**) Cells were treated with PHE for 48 h and stained with annexin V/PI. (**B**,**C**) Statistical analysis of the apoptosis ratio of MDA-MB-231 and MCF-7 cells after PHE treatment. All data are expressed as mean ± SD of three independent experiments. * *p* < 0.05, ** *p* < 0.01, *** *p* < 0.001 compared to the control of each cell line.

To determine whether the cytotoxic effect of PHE was associated with apoptosis, annexin V-FITC/PI double staining was used to determine the number of apoptotic cells by flow cytometry analysis. Phosphatidylserine (PS) is a key biomarker of early apoptosis and is translocated to the extracellular domain from the cytosolic portion of the membrane, which is identified by annexin V-FITC staining. As apoptosis proceeds further, the cell membrane is destroyed and the PI that goes into the nucleus eventually stains the DNA. The MDA-MB-231 cell line displayed higher sensitivity than the MCF-7 cell line in response to the apoptosis induction of PHE at concentrations of 1, 3, 10, and 30 µg/mL for 48 h. Early apoptosis increased to 5.6 ± 1.33%, 2.36 ± 0.15%, 7.06 ± 0.96%, 31.1 ± 0.51%, and 37.4 ± 1.7%; later apoptosis also increased to 6.06 ± 0.35%, 5.56 ± 0.15%, 8.93 ± 0.66%, 18.4 ± 1.67%, and 23.53 ± 0.23% (Figure 3A). The results indicated that differences in apoptosis rates occurred in MDA-MB-231 and MCF-7 cells treated with PHE (Figure 3B,C).

### 3.4. Effect of M47 on the Levels of Apoptosis-Related Proteins

**Figure 4:**
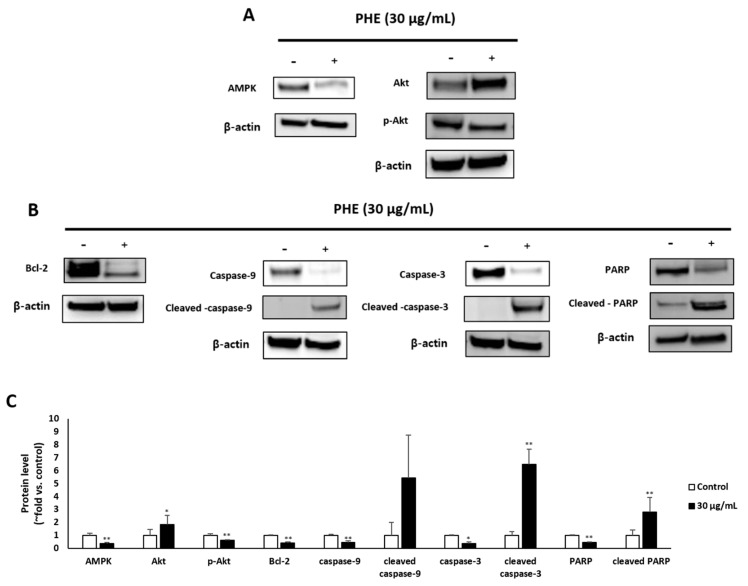
*Physconia hokkaidensis* methanol extract (PHE) induces apoptotic cell death of MDA-MB-231 cells. MDA-MB-231 cells were treated with 30 µg/mL of PHE for 48 h. (**A**,**B**) Apoptotic markers were analyzed by Western blotting and β-actin was used as a protein loading control. (**C**) The level of apoptotic markers in lichen-extract-treated MDA-MB-231 cells was analyzed. Protein levels were normalized to the control group. (−) means “without treatment of PHE”, (+) means “ with treatment of PHE”, * *p* < 0.05, ** *p* < 0.01 compared to the control of each cell line.

The transcriptional levels of various pro- and antiapoptotic proteins in PHE-treated MDA-MB-231 cells were examined by Western blotting. The activation of AMP-activated protein kinase (AMPK) and *p*-Akt leads is well known to mediate antiapoptotic effects, whereas deactivation of the AMPK pathway results in elevated apoptosis levels. The treatment of MDA-MB-231 with PHE at 30 µg/mL for 48 h showed significantly decreased protein levels of AMPK and *p*-Akt (Figure 4A). Next, Western blotting was further performed to analyze the effect of PHE treatment on antiapoptotic and apoptotic proteins. The treatment with PHE for 48 h induced the decrease of the transcriptional level of antiapoptotic proteins such as Bcl-2 but increased levels of key apoptotic proteins, including cleaved caspase-9/-3 and cleaved PARP (Figure 4B).

## 4. Discussion

Cancer treatment still has many unmet needs and requires new therapeutic agents. New and efficient drugs against cancer should be developed for public health, and for this purpose, the identification of new substances with anticancer activity is essential in the development of anticancer drugs. Many reports have mentioned that lichens have potential as a source of biologically active substances for the development of anticancer agents [14,15,16]. 

A few lichen species have been reported to have nonselective cytotoxicity against breast cancer cells including MDA-MB-231, triple-negative breast cancer (TNBC) cells, and MCF-7 cells expressing ER and PR but not HER-2. Lichen species, such as *Xanthoparmelia somloensis* (Gleyn.) Hale, *Usnea intermedia* (A. Massal.) Jatta, *Bryoria capillaris* (Ach.) Brodo & D. Hawksw, and *Lobaria pulmonaria* (L.) Hoffm, have an antiproliferative effect on MDA-MB-231 and MCF-7 cells, as well lung cancer cells, by inducing apoptosis [20]. Nanoparticles manufactured with lichens such as *Xanthoria parietina* and *Flavopunctelia flaventior* showed antibacterial and cytotoxic activity on both MDA-MB-231 and MCF-7 cells [21]. A lichen, *Parmelia sulcata* Taylor, exhibited promising anticancer activity against breast cancer cells and a genotoxic effect on human lymphocytes [22]. In addition to lichen extracts, atranorin, a metabolite isolated from lichens including *Stereocaulon cacspitorim*, *Everniastrum vexans*, and *Parmatrema* species, exhibited anti-breast cancer activity on MDA-MB-231 and MCF-7 cells through an effect on Akt activity [23]. 

However, the cytotoxicity of PHE was evaluated in various cancer cells, and we identified an anticancer effect of the methanol extract of *Physconia hokkadensis* (PHE). It showed remarkable cytotoxicity against MDA-MB-231 cells, triple-negative breast cancer (TNBC) cells, but not on MCF-7 (Figure 1 and Table 1). Then, the study was continued by identifying differences in the effect of PHE on TNBC MDA-MB-231 and MCF-7. Cell cycle analysis and annexin V/PI staining were performed to identify the mechanism of action of cytotoxicity from PHE. The MDA-MB-231 treated with PHE showed the arrest of sub-G1 (Figure 2), implying PHE is able to induce apoptosis because the sub-G1 fraction of DNA-labeled cells is usually related to apoptotic cells and because DNA fragmentation is a feature of cell death. During apoptosis, the genome DNA is cut into small fragments of approximately 180 bp (and multiples of it) each and placed below G1/G0 peak after dyeing with propidium iodide [24,25]. Annexin V/PI staining showed a dose-dependent increase in MDA-MB-231 but a consistent level of staining in MCF-7 (Figure 3). During apoptosis, lipid asymmetry in cell membranes is lost and phosphatidylserine (PS) is exposed on the outer leaflet of the plasma membrane, which is the “eat me” signal for apoptosis. Annexin V, a 36-kDa calcium-binding protein, can bind to the extracellular portion of PS. Fluorescently labeled annexin V is generally used to detect PS that is exposed on the outside of cell membrane in early/mid-stage apoptosis and to distinguish apoptotic and necrotic cells by flow cytometry [26]. Most drugs used to treat carcinoma are associated with the activation of apoptosis through the mitochondrial pathway [27,28,29]. Changes in mitochondrial membrane potential are considered an important indicator of the onset of apoptosis [30,31,32,33]. The altered membrane potential consequently initiates the outflow of cytochrome c, which activates caspase-3 and caspase-9 [30,31,32,33]. As shown in Figure 4B,C, PHE induced the increase of levels of cleaved caspase-3/9 and cleaved PARP, supporting the idea that cytotoxicity of PHE is induced via apoptosis. In contrast to apoptotic proteins such as cleaved caspase-3/9, Bcl-2 protein is a representative oncogenic and antiapoptotic protein, and it acts as a radical-scavenging agent by scavenging radicals generated by being contained in the membranes of the nucleus, endoplasmic reticulum, and mitochondria [34,35]. In addition to Bcl-2, AMPK and Akt are proliferative proteins related to the oncogenic signaling pathway in the progression of cancer and are potential therapeutic targets for cancer treatment [36,37]. Akt, a downstream target of AMPK, regulates the proliferation, survival, and growth of many human cancer cells [38]. It has been reported that Akt on phosphorylation acts as an antiapoptotic molecule and prevents cells from undergoing apoptosis [39]. As shown in Figure 4A,B, Western blotting of MDA-MB-231 treated with 30 µg/mL of PHE revealed that antiapoptotic biomarkers, including Bcl-2, AMPK, and *p*-Akt, were significantly reduced. Taken together, this study supports that the cytotoxic activity of PHE on MDA-MB-231 cells is clearly derived from the induction of apoptosis. 

## 5. Conclusions

In summary, the present study documented the selective cytotoxic effect of PHE in MDA-MB-231 TNBC cells compared to MCF-7 breast cancer cells with expression of ER and PR. The cell cytotoxic activity of the PHE on MDA-MB-231 cells is probably derived from the cell cycle arrests and apoptosis. These results suggest that the PHE can be a potential candidate as a hit compound to develop a therapeutic agent for the treatment of MDA-MB-231 breast cancer. Further studies are still needed to explore its exact mechanism of action and assess its therapeutic efficacy as a candidate for the development of an agent against cancer. 

## Figures and Tables

**Table 1:** Effect of PHE on the viability of various cell lines.

	IC_50_ Value (µg/mL)
	PHE
MDCK (Madin–Darby canine kidney cells)	>100
HL-60 (Human acute promyelocytic leukemia cells)	11.3 ± 0.4
HeLa (Human cervix adenocarcinoma cells)	21.1 ± 2.4
A549 (Human lung carcinoma cells)	7.6 ± 3.3
Hep G2 (Human hepatoblastoma cells)	>30
AGS (Human gastric carcinoma cells)	22.6 ± 1.2
MDA-MB-231 (Human breast adenocarcinoma cells)	23.7 ± 1.2
MCF-7 (Human breast adenocarcinoma cells)	>100

Seven cancer cell lines and a normal cell line were treated with PHE for 24 h. The culture supernatant was removed and cell counting kit-8 was added. Viability was quantified using a microplate reader. Data are presented as 50% inhibitory concentration (IC_50_, µg/mL). Values are presented as mean ± SD of three independent experiments.

## Supplementary Materials

The following are available online at https://www.mdpi.com/2309-608X/7/3/188/s1, Figure S1: Effect of PHE on the viability of MDCK and MCF-7, Figure S2: HPLC chromatogram profiles of *Physconia hokkaidensis* methanol extract, Table S1: Integration of chromatogram.
