# Supplementary material for: Induction of Apoptosis in MDA-MB-231 Cells Treated with the Methanol Extract of Lichen Physconia hokkaidensis
Source: J Fungi (Basel). 2021 Mar 5;7(3):188. doi: 10.3390/jof7030188 (PMC8000577; doi:10.3390/jof7030188)
Supplement: Supplementary file 1 [file jof-07-00188-s001.pdf]

Supplementary Material

Induction of apoptosis in MDA-MB-231 cells treated with the methanol extract of lichen *Physconia hokkaidensis*

Ji-In Noh [1], Seul-Ki Mun [1], Eui Hyeon Lim [1], Hangun Kim [1], Dong-Jo Chang [1], Jae-Seoun Hur [2] and Sung-Tae Yee [1,*]

[1] Department of Pharmacy, Sunchon National University, 255 Jungang-Ro, Suncheon 549-742, Korea; nji8009@naver.com (J.-I.N.); motomoto1210@naver.com (S.-K.M.); sksms147zld@naver.com (E.H.L.); hangunkim@sunchon.ac.kr (H.K.); djchang@scnu.ac.kr (D.-J.C.)

[2] Department of Environmental Education, Korea Lichen Research Institute, Sunchon National University, Suncheon 549-742, Korea; jshur1@sunchon.ac.kr

* Correspondence: sungtae@sunchon.ac.kr; Tel.: +82-61-750-3752; Fax: +82-61-750-3708

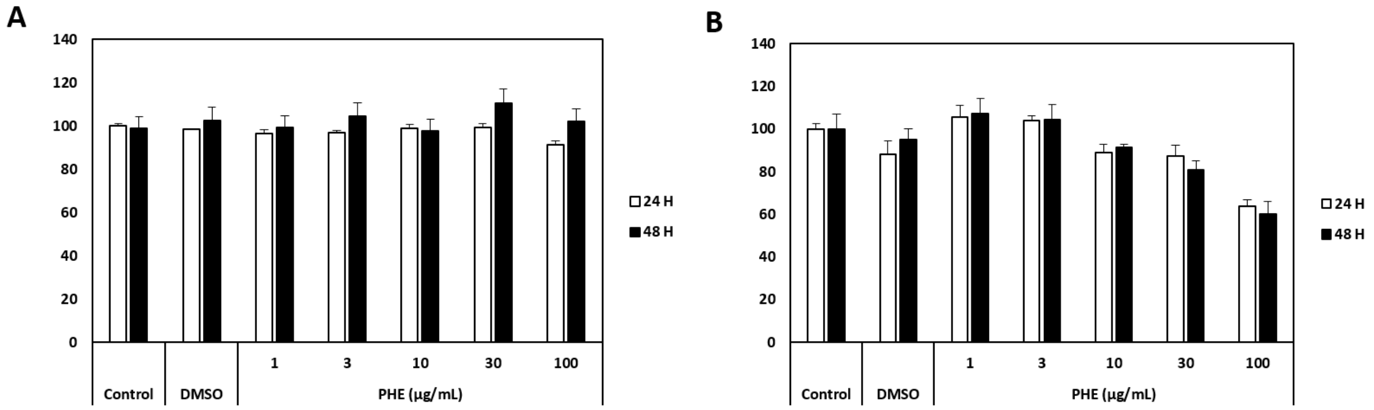

Figure S1. Effect of PHE on the viability of MDCK and MCF-7. (**A**), (**B**) MDCK and MCF-7 were treated with the PHE (1, 3, 10, 30 and 100 μg/mL) for 24 h and 48 h. The culture supernatant was removed and cell counting kit-8 was added. Viability was quantified using a microplate reader.

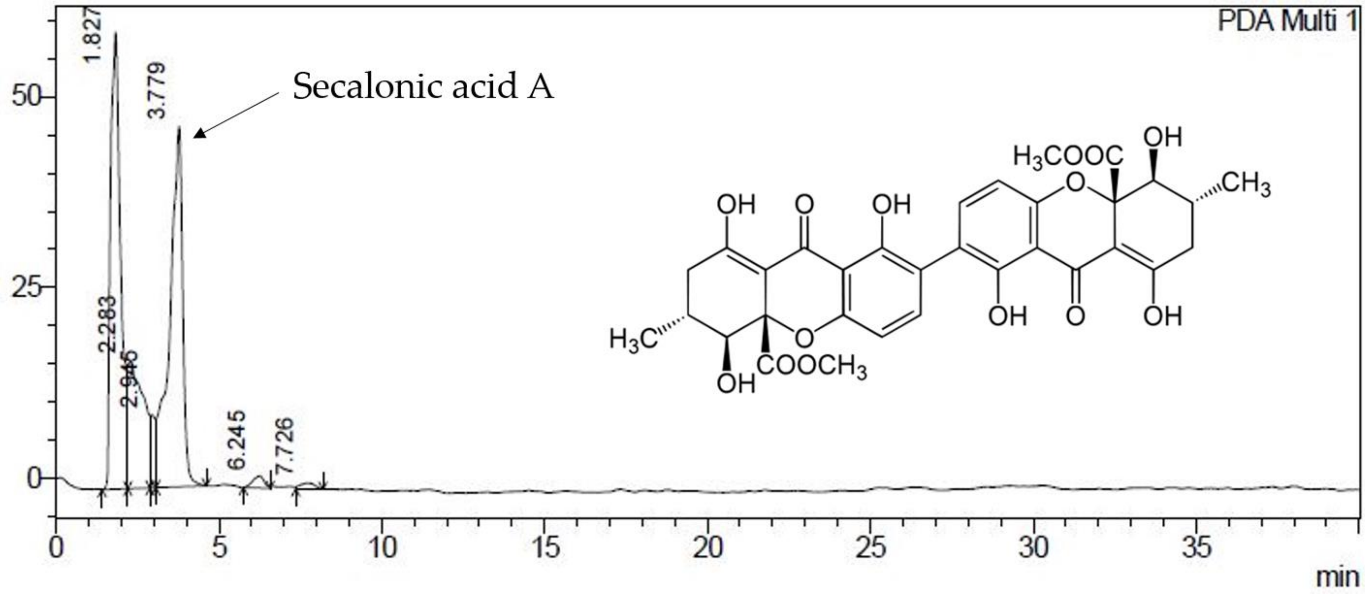

Figure S2. HPLC chromatograms profiles of *Physconia hokkaidensis* methanol extract. HPLC spectrum acquired at 254 nm together with the chemical structures of their predominant constituents of methanol extract from *Physconia hokkaidensis* lichen (PH).

Table S1. Integration of chromatogram

Peak#	Ret. Time	Name	Area	Area%
1	1.827	RT:1.1827	13.7880	39.274
2	2.283	RT:2.283	545742	16.388
3	2.945	RT:2.945	101279	3.041
4	3.779	Secalonic acid A	1311348	39.378
5	6.245	RT:6.245	39153	1.176
6	7.726	RT:7.726	24736	0.743
Total			3330137	100.000
